# Performance of clinician prediction of survival in oncology outpatients with advanced cancer

**DOI:** 10.1371/journal.pone.0267467

**Published:** 2022-04-21

**Authors:** Yu Jung Kim, Seok Joon Yoon, Sang-Yeon Suh, Yusuke Hiratsuka, Beodeul Kang, Si Won Lee, Hong-Yup Ahn, Koung Jin Suh, Ji-Won Kim, Se Hyun Kim, Jin Won Kim, Keun-Wook Lee, Jee Hyun Kim, Jong Seok Lee

**Affiliations:** 1 Division of Hematology and Medical Oncology, Department of Internal Medicine, Seoul National University College of Medicine, Seoul National University Bundang Hospital, Seongnam, South Korea; 2 Department of Family Medicine, Chungnam National University Hospital, Daejeon, South Korea; 3 Department of Family Medicine, Dongguk University Ilsan Hospital, Goyang, South Korea; 4 Department of Medicine, Dongguk University Medical School, Seoul, South Korea; 5 Department of Palliative Medicine, Takeda General Hospital, Aizu Wakamatsu, Japan; 6 Department of Palliative Medicine, Tohoku University School of Medicine, Sendai, Japan; 7 Division of Medical Oncology, Bundang Medical Center, CHA University, Seongnam, South Korea; 8 Division of Medical Oncology, Department of Internal Medicine, Yonsei University College of Medicine, Yonsei Cancer Center, Seoul, South Korea; 9 Department of Statistics, Dongguk University Medical School, Seoul, South Korea; University of California San Francisco, UNITED STATES

## Abstract

**Background:**

We aimed to investigate the performance of clinician prediction of survival (CPS) and the association between CPS and the prognostic confidence of clinicians in ambulatory medical oncology outpatients.

**Methods:**

Eight medical oncologists estimated the expected survival of their patients in a prospective cohort study. They were asked to provide a temporal type of CPS in weeks, together with their level of confidence for each prediction (0–100%). We analyzed the accuracy of CPS, the association between CPS and the prognostic confidence, and the characteristics of patients showing inaccurate CPS.

**Results:**

A total of 200 advanced cancer patients were enrolled and the median overall survival was 7.6 months. CPS was accurate in 67 (33.5%) patients, underestimated in 87 (43.5%), and overestimated in 46 (23.0%). The overall accuracy of CPS for 12-week, 24-week, 36-week, and 48-week survival was 83.0%, 72.0%, 75.5%, and 74.0%, respectively. The specificity was highest for 12-week survival (90.2%) and the sensitivity was highest for 48-week survival (96.1%). The sensitivity of 12-week CPS was 51.4% and the area under the receiver operating characteristic (AUROC) curve was 0.79 (95% confidence interval [CI] 0.71–0.87). The prognostic confidence of clinicians was not significantly associated with the accuracy of prediction (*P* = 0.359). Patients with overestimated survival had significantly poorer global health status and physical/role/emotional functioning in the European Organization for Research and Treatment of Cancer Quality of Life Questionnaire Core 30 (EORTC QLQ-C30). Additionally, they showed significantly higher levels of fatigue, nausea/vomiting, pain, dyspnea, and loss of appetite.

**Conclusion:**

The overall accuracy of CPS in predicting 12-week to 48-week survival was high in medical oncology outpatients. However the sensitivity of 12-week CPS was low and prognostic confidence was not associated with the accuracy of CPS. Patients with overestimated CPS showed poorer quality of life and higher symptom burden.

## Introduction

Accurate prediction of survival in advanced cancer patients is crucial for various reasons. Better prognostication may allow patients to make better decisions regarding their medical care near the end of life [1, 2]. Thus physicians consistently try to estimate the remaining survival of their patients using clinician prediction of survival (CPS) or validated prognostic scores [1, 3].

CPS is the most common method to predict survival in advanced cancer patients [4, 5]. Although there is a great deal of controversy regarding the accuracy of CPS, it is considered an independent prognostic factor, along with other known prognostic factors. CPS has a significant correlation with actual survival in terminally ill cancer patients, with a correlation coefficient of 0.2 to 0.65 [1]. However, it is well known that physicians tend to overestimate survival [6, 7]. Physicians can be overly optimistic because accurate prognostication itself is difficult and they want to preserve the hopes of patients. One study reported that experienced physicians and physicians who do not have long-term relationships with the patient may be better at predicting prognosis [8].

In addition, CPS is known to be more accurate for short-term predictions than for long-term predictions [1]. The variability of actual survival increases with longer CPS [6]. Indeed, the vast majority of studies on CPS were performed in terminally ill cancer patients with an expected survival of less than 2 months [5]. There have been scarce studies on the accuracy of CPS in ambulatory advanced cancer patients with prognoses of months to years [9, 10]. One large prospective cohort study from Japan investigated the accuracy of CPS in 2,036 patients in various groups, including hospital palliative care teams, palliative care units, home palliative care services, and active chemotherapy [10]. Although this study reported a high correlation coefficient of 0.70 between CPS and actual survival in patients receiving active chemotherapy, the median survival was only 34 days (95% confidence interval [CI] 29–39) in these patients.

In this study, we aimed to investigate the performance of CPS and the association between prognostic confidence and accuracy of CPS in ambulatory medical oncology outpatients. We hypothesized that CPS would not be accurate in predicting survival times longer than 12 weeks in advanced cancer patients. Prognostic confidence was expected to increase the accuracy of CPS.

## Materials and methods

### Patients

This study was part of a prospective cohort study aimed at developing a prognostic model in medical oncology outpatients [11]. The prognostic model predicts 3-month survival to facilitate palliative care referral at least 3 months before death. We enrolled advanced cancer patients treated at a comprehensive cancer center of a university hospital from March 2016 to January 2019. Patients were eligible if they had a diagnosis of advanced cancer, when their oncologists estimated their survival to be less than a year and were 18 years or older. We defined advanced cancer as a metastatic or recurrent disease or progressive locally advanced disease not amenable to curative treatment. Patients were excluded if they had hematologic malignancies, were expected to survive less than a month, and were not able to communicate. Written informed consent was obtained from each patient before enrollment. The protocol was approved by the Institutional Review Board (IRB) of Seoul National University Bundang Hospital (IRB number: B-1601/332-302).

### Data collection

Medical oncologists were asked to estimate CPS with a temporal question, “How many weeks will this patient have?” They were also asked to provide their level of confidence for each prediction in percentage (0% [not at all] to 100% [full confidence]). The age, sex, years of clinical experience, and palliative care training experience of each oncologist were collected. The patients were interviewed face to face by a clinical research nurse after enrollment. Patient performance status was assessed by the Eastern Cooperative Oncology Group (ECOG) performance status and Karnofsky Performance Scale (KPS). All patients were asked to complete the European Organization for Research and Treatment of Cancer Quality of Life Questionnaire Core 30 (EORTC QLQ-C30) version 3.0 [12]. The EORTC QLQ-C30 consists of multi-item scales including functional scales (physical, role, emotional, cognitive, and social), symptom scales (fatigue, pain, nausea/vomiting, pain, dyspnea, insomnia, appetite, constipation, diarrhea, and financial difficulties), and a global health status/quality of life (QoL) scale. All of the scales and single-item measures range in score from 0 to 100. High scores for the functional scale and the global health status/QoL represent a high/healthy level of functioning and high QoL. On the contrary, high scores in the symptom scale mean high symptom burden. We obtained demographic data, clinical information, and laboratory test results from the electronic medical records of patients.

### Statistical analysis

Descriptive analyses were performed to summarize the baseline characteristics of the patients. The CPS was considered accurate if it fell within ±33.3% of the actual survival as previously described in other studies [8, 13, 14]. The median survival and 95% CI were calculated using the Kaplan-Meier method. Survival time was observed from enrollment into this study until death. Live cases at the end of the study were dealt with as censored data. The comparison of survival outcomes was performed using log-rank tests. To assess the discriminatory ability of the CPS, we calculated the sensitivity, specificity, positive predictive value (PPV), negative predictive value (NPV), overall accuracy, and area under the receiver operating characteristic (AUROC) curve. The comparison of EORTC QLQ-C30 scores among different CPS groups (accurate, underestimated, and overestimated) was performed using one-way analysis of variance (ANOVA). All tests were two-sided, and *P*-values <0.05 were considered significant. All analyses were performed using IBM SPSS Statistics for Windows, version 26.0 (IBM Corp., Armonk, NY, USA).

## Results

A total of 200 advanced cancer patients were enrolled in this study. With a median follow-up period of 7.7 months (range, 0.5–36.8), the median overall survival was 7.6 months (95% CI 1.3–33.3), and 159 patients had died at the time of analysis. The predictions of 8 oncologists were distributed as S1 Fig. CPS was accurate in 67 (33.5%) patients, underestimated in 87 (43.5%), and overestimated in 46 (23.0%). A total of 64 patients (32%) were alive after a year. Baseline patient characteristics according to prognostic accuracy are summarized in Table 1.

**Table 1:** Baseline patient characteristics (n = 200).

Characteristics	All patients, n = 200 (%)	Accurate^a^, n = 67 (%)	Underestimated, n = 87 (%)	Overestimated, n = 46 (%)	*P*-value^b^
Age, years (mean ± SD)	64.4±11.6	65.0±10.9	63.7±12.2	65.1±11.4	0.703
Sex					0.460
Female	72 (36.0)	28 (41.8)	28 (32.2)	16 (34.8)	
Primary cancer site					
Lung	67 (33.5)	21 (31.3)	25 (28.7)	21 (45.7)	0.130
Stomach	20 (10.0)	7 (10.4)	9 (10.3)	4 (8.7)	0.945
Colon/Rectal	28 (14.0)	11 (16.4)	10 (11.5)	7 (15.2)	0.658
Breast	18 (9.0)	8 (11.9)	6 (6.9)	4 (8.7)	0.554
Gynecologic	4 (2.0)	3 (4.5)	0 (0.0)	1 (2.2)	0.144
Liver/Biliary tract/Pancreas	8 (4.0)	2 (3.0)	4 (4.6)	2 (4.3)	0.871
Genitourinary	29 (14.5)	8 (11.9)	17 (19.5)	4 (8.7)	0.184
Others	26 (13.0)	7 (10.4)	16 (18.4)	3 (6.5)	0.115
ECOG performance status					0.048
0	7 (3.5)	3 (4.5)	4 (4.6)	0 (0.0)	
1	125 (62.5)	41 (61.2)	61 (70.1)	23 (50.0)	
2	55 (27.5)	17 (25.4)	17 (19.5)	21 (45.7)	
3	13 (6.5)	6 (9.0)	5 (5.7)	2 (4.3)	
Karnofsky Performance Status					0.035
50	5 (2.5)	2 (3.0)	2 (2.3)	1 (2.2)	
60	16 (8.0)	5 (7.5)	5 (5.7)	6 (13.0)	
70	68 (34.0)	20 (29.9)	24 (27.6)	24 (52.2)	
80	86 (43.0)	32 (47.8)	40 (46.0)	14 (30.4)	
90	25 (12.5)	8 (11.9)	16 (18.4)	1 (2.2)	

Abbreviations: ECOG, Eastern Cooperative Oncology Group; n, number; SD, standard deviation.

^a^Accurate estimation was defined as clinician prediction of survival being within ±33.3% of actual survival.

^b^*P*-values were obtained by comparison among different accuracy groups with one-way analysis of variance (ANOVA) or chi-square test.

Eight medical oncologists participated in this study. The characteristics of the clinicians are shown in Table 2. Half of the clinicians were female, and most of them had more than 10 years of clinical experience. Three clinicians (37.5%) had palliative care training and dedicated palliative care experience.

**Table 2:** Clinician characteristics (n = 8).

Characteristics	Medical oncologists (n = 8)
Age, years, median (range)	39 (33–45)
Female	4 (50%)^a^
Years of clinical experience, median (interquartile range)	14.0 (9.5–17.0)
Palliative care training	3 (37.5%)

^a^Descriptive data were presented as numbers (%).

The overall accuracy of CPS for predicting 12-week, 24-week, 36-week, and 48-week survival was 83.0%, 72.0%, 75.5%, and 74.0%, respectively (Table 3). The specificity was highest for 12-week survival (90.2%) and the sensitivity was highest for 48-week survival (96.1%). The sensitivity of 12-week CPS was 51.4% and the AUROC curve was 0.79 (95% CI 0.71–0.87). The AUROC curves of CPS for 24-week, 36-week, and 48-week survival were 0.81 (95% CI 0.75–0.87), 0.83 (95% CI 0.77–0.88), and 0.82 (95% CI 0.76–0.88), respectively.

**Table 3:** Accuracy of clinician prediction of survival^a^.

	Prevalence^b^ (%)	Sensitivity	Specificity	PPV	NPV	Overall accuracy^c^ (%)	AUROC
		(%)	(%)	(%)	(%)		(95% CI)
12-week survival	37/200 (18.5)	51.4	90.2	54.3	89.1	83.0	0.79 (0.71–0.87)
24-week survival	76/200 (38.0)	84.2	64.5	59.3	87.0	72.0	0.81 (0.75–0.87)
36-week survival	110/200 (55.0)	82.7	66.7	75.2	75.9	75.5	0.83 (0.77–0.88)
48-week survival	128/200 (64.0)	96.1	34.7	72.4	83.3	74.0	0.82 (0.76–0.88)

Abbreviations: AUROC, area under the receiver operating characteristic curve; CI, confidence interval; NPV, negative predictive value; PPV, positive predictive value.

^a^All 200 patients have been followed until their death or the end of this study. The median follow-up time (including dead and alive cases) was 7.7 months (min 0.5—max 36.8 months). Among living patients, only one patient was followed for 40 weeks. Except for this patient, all living patients were followed for more than 48 weeks.

^b^Prevalence was defined as death events in each time frame per total study population.

^c^Overall accuracy was calculated as the arithmetic means of sensitivity, specificity, PPV, and NPV.

**Fig 1:**
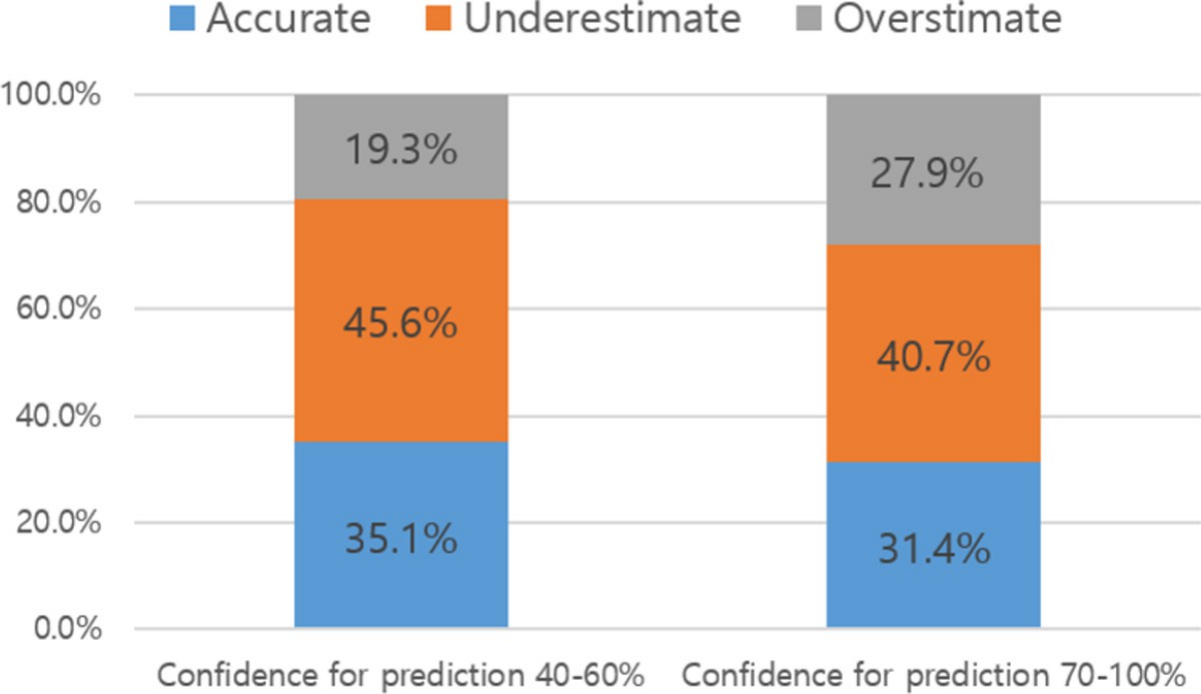
Accuracy of clinician prediction of survival by prognostic confidence in prediction. ^a^*P*-value was 0.359 by chi-square test. ^b^Accurate estimation was defined as clinician prediction of survival being within ±33.3% of actual survival. ^c^Clinicians’ prognostic confidence was measured by a percent scale from 0% (not at all) to 100% (full confidence).

The prognostic confidence of clinicians was not significantly associated with the accuracy of CPS (*P* = 0.359) (Fig 1). CPS was accurate in 35.1% of clinicians, with a 40–60% confidence level and 31.4% of clinicians with a 70–100% confidence level. The correlation coefficients between CPS and actual survival time were 0.60 (*P*<0.001) and 0.62 (*P*<0.001) in clinicians with a 40–60% confidence level and a 70–100% confidence level, respectively.

**Fig 2:**
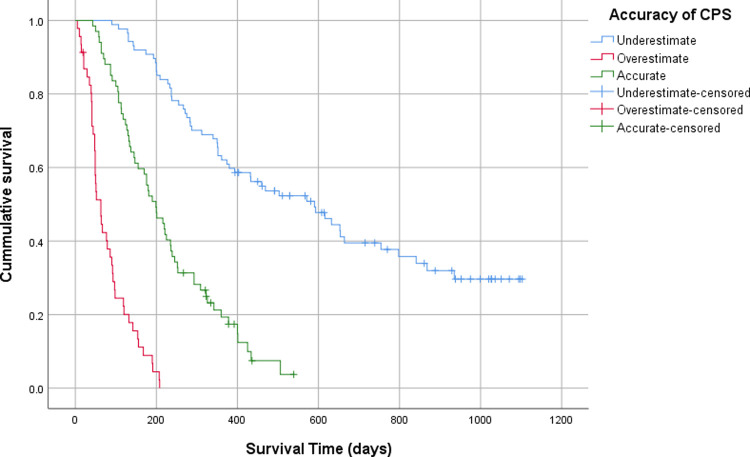
Survival according to the accuracy of clinician prediction of survival. Patients with accurate clinician prediction of survival (CPS), underestimated CPS, and overestimated CPS had median survival times of 6.6 months (95% CI 5.1–8.2), 19.7 months (95% CI 13.6–25.8), and 2.1 months (95% CI 1.5–2.7), respectively. The differences in survival in the three groups were all significant (accurate CPS vs. underestimated CPS: *P*<0.001; accurate CPS vs. overestimated CPS: *P*<0.001) by the log-rank test.

Patients with accurate CPS, underestimated CPS, and overestimated CPS had median survival times of 6.6 months (95% CI 5.1–8.2), 19.7 months (95% CI 13.6–25.8), and 2.1 months (95% CI 1.5–2.7), respectively (Fig 2). The differences in survival in the three groups were all significant (accurate CPS *vs*. underestimated CPS: *P*<0.001; accurate CPS *vs*. overestimated CPS: *P*<0.001) by the log-rank test.

Table 4 depicts the association between the accuracy of the CPS and EORTC QLQ-C30 scores. Patients with overestimated survival had significantly poorer global health status/QoL scores (*P* = 0.004) and poorer physical (*P* = 0.001), role (*P* = 0.003), and emotional (*P* = 0.001) functioning scores. In addition, they showed significantly higher levels of fatigue (*P* = 0.014), nausea/vomiting (*P* = 0.001), pain (*P*<0.001), dyspnea (*P*<0.001), and loss of appetite (*P*<0.001) compared with the other two groups of patients.

**Table 4:** Association between the accuracy of clinician prediction of survival and mean EORTC-QLQ-C30 scores.

Scores^a^	Accurate,	Underestimated,	Overestimated,	*P*-value^b^
	n = 67 (SD)	n = 87 (SD)	n = 46 (SD)	
**Global health status/Quality of life (QoL)**				
Global health/QoL	51.24 (20.98)^1c^	49.52 (20.23)^1^	39.31 (16.64)^2^	0.004
**Functional scales**				
Physical functioning	57.71 (22.19)^1^	62.30 (23.77)^1^	46.96 (20.58)^2^	0.001
Role functioning	54.48 (32.25)^1^	58.05 (29.30)^1^	39.86 (23.70)^2^	0.003
Emotional functioning	80.10 (20.00)^1^	74.90 (22.25)^1^	64.49 (21.33)^2^	0.001
Cognitive functioning	72.64 (22.04)	71.84 (20.70)	67.75 (22.88)	0.466
Social functioning	61.94 (28.98)	59.39 (32.32)	48.55 (29.15)	0.060
**Symptom scales/item**				
Fatigue	45.94 (27.35)^1, 2^	43.93 (25.30)^1^	57.73 (26.46)^2^	0.014
Nausea/Vomiting	12.94 (23.54)^1^	13.79 (19.22)^1^	27.90 (27.90)^2^	0.001
Pain	36.07 (32.51)^1^	30.08 (29.40)^1^	52.54 (27.66)^2^	<0.001
Dyspnea	29.35 (28.14)^1^	26.82 (30.85)^1^	49.28 (34.24)^2^	<0.001
Insomnia	35.32 (35.71)	36.78 (30.93)	42.03 (31.77)	0.546
Appetite	38.31 (31.92)^1^	42.15 (33.12)^1^	68.12 (30.60)^2^	<0.001
Constipation	27.36 (36.20)	23.37 (29.25)	35.51 (33.26)	0.128
Diarrhea	7.96 (18.43)	13.79 (24.67)	10.87 (21.15)	0.264
Financial difficulties	33.83 (31.51)	31.80 (32.91)	39.86 (32.67)	0.391

Abbreviations: EORTC-QLQ-C30, European Organization for Research and Treatment of Cancer Quality of Life Questionnaire Core 30; SD, standard deviation.

^a^Higher scores mean better global health status/quality of life, better functional status and worse symptoms. Each score ranges from 0 to 100.

^b^*P*-values were obtained by comparison among different accuracy groups with one-way analysis of variance (ANOVA).

^c^Superscripts (1, 2) indicate significant differences by post-hoc test.

## Discussion

We found that the overall accuracy of CPS in predicting 12-week to 48-week survival in ambulatory medical oncology outpatients was higher than expected in this preplanned analysis of a prospective cohort study. The overall accuracy of 12-week CPS was 83.0%, and the specificity was 90.2%. However, the sensitivity of 12-week CPS was limited to 51.4%. This low sensitivity may show the limitation of using CPS as a sole prognostic factor to predict 12-week survival. To improve the accuracy of CPS, clinicians may need to combine other prognostic factors or scores [1]. We are currently developing a prognostic model to predict 3-month survival in oncology outpatients.

In terms of the accuracy of temporal CPS (defined as ±33.3% of the actual survival), 67 patients (33.5%) were accurately predicted in our study. The percentage of accurate estimates is reported to be 23–78% in various studies [15]. However, direct comparison of the results is not possible because of heterogeneous categories and definitions used in different studies. The result of our study is similar to previous studies that used the same definition as ours. In one study, the accuracy of temporal CPS was 32% for physicians and 18% for nurses [14]. In another study, 35% of survival estimations were classified as accurate for palliative care physicians in 2,036 patients [10]. Another study that included only CPS by medical oncologists as our study reported that CPS was accurate in 29% of patients [9].

Unexpectedly, underestimated CPS was more frequent than overestimated CPS in our study. It is well known that CPS is overly optimistic in terminally ill patients [6, 8]. However, the nature of CPS in ambulatory medical oncology outpatients has not been clearly clarified. A recent systematic review reported that clinicians in 5 studies underestimated patient survival out of 15 studies [7]. In a study on CPS by medical oncologists, the median estimated survival time was 11 months, while the median actual survival time was 9 months [9]. The study excluded patients with an expected survival of less than 3 months, and 61% of patients lived longer than the estimates of oncologists. According to a study that compared the CPS of medical professionals in advanced cancer patients with brain metastases, neurosurgeons and radiation oncologists were generally more optimistic [16]. Interestingly, only medical oncologists underestimated survival. The reasons for the higher frequency of underestimation in medical oncologists are unknown. However, because they play a major role in the care planning of metastatic cancer patients with limited survival, the negative consequences of overestimation would be particularly distressing to medical oncologists. Additionally, a consistent rise in patients responding to newer chemotherapeutic agents may have contributed to longer survival, which lies behind the underestimation. In our study, 32% of patients lived longer than a year, despite the inclusion criteria of expected survival less than a year. Interestingly, one study reported that CPS tended to be optimistic in patients with short-lived survival (≤6 months) and pessimistic in patients with longer survival (≥9 months) [17]. There were no significant differences in the direction of survival prediction between physicians, nurses, and radiation therapists in that study.

We hypothesized that the prognostic confidence of clinicians would have a positive association with the accuracy of CPS. Unlike our assumption, there was no significant association between the prognostic confidence and accuracy of CPS in this study. Clinicians with a lower level of prognostic confidence (40–60%) showed accuracy similar to clinicians with a higher level of prognostic confidence (70–100%). The correlation coefficient between CPS and actual survival time in the lower confidence group was 0.60 (*P*<0.001) and that of the higher confidence group was 0.62 (*P*<0.001). Thus, the correlation coefficients were similarly high in the two groups. Previous studies have reported that the CPS of experienced physicians was more accurate than that of less experienced physicians, but this may not mean that experienced physicians have more prognostic confidence [8, 18]. In our study, all medical oncologists were highly experienced, and their prognostic confidence might have been influenced by modest personalities. Further studies are needed to investigate the association between prognostic confidence and the accuracy of CPS.

In our study, patients with overestimated CPS showed an absolutely and significantly short median survival of 2.1 months compared with the other two groups of patients. Furthermore, these patients had significantly poorer global health/QoL, poorer functioning sores, and higher symptom scores according to the EORTC QLQ-C30. It is not surprising that patients near the end-of-life suffer from poor QoL and high symptom burdens. However, physicians should not forget that poor QoL and high symptom burden are indicators of poor prognosis. As described in previous studies, physical symptoms such as nausea/vomiting, dyspnea, and fatigue may reflect the consequences of cancer cachexia and terminal diseases [19]. It is not easy for physicians to recognize wide range of symptoms instantly in busy clinical setting. Thus there is a possibility that patients’ high symptom burden might have been overlooked at the time of prediction in our study. In this regard, we suggest routine symptom screening using symptom assessment tools in everyday practice. We believe proactive symptom screening in medical oncology outpatients can improve the accuracy of CPS.

The strengths of our study may include the prospective design, investigation of CPS in medical oncology outpatients with a relatively longer survival compared with terminally ill patients receiving hospice care, and demonstration of high overall accuracy of CPS in this population. However, our study was conducted in a single tertiary cancer center in Korea, which may limit the general applicability of the study results. Another limitation may include the small number of participating medical oncologists (n = 8). Finally, the main eligibility criterion was a life expectancy of less than a year. This might have biased the CPS towards underestimation.

## Conclusions

In conclusion, the survival estimates of medical oncologists were not highly accurate (33.5%) in this study, but the overall accuracy of CPS in predicting 12-week to 48-week survival was high (72–83%). Prognostic confidence was not associated with the accuracy of CPS. Finally, patients with overestimated CPS showed poorer QoL, higher symptom burden, and significantly poorer survival.

## Supporting information

S1 Fig Distribution of clinician prediction of survival (CPS, weeks). ^a^Frequency is expressed as percentage (%).
(TIF)

S1 Data (XLSX)
