# Supplementary figures and images for: Performance of clinician prediction of survival in oncology outpatients with advanced cancer
Source: PLoS One. 2022 Apr 21;17(4):e0267467. doi: 10.1371/journal.pone.0267467 (PMC9022805; doi:10.1371/journal.pone.0267467)

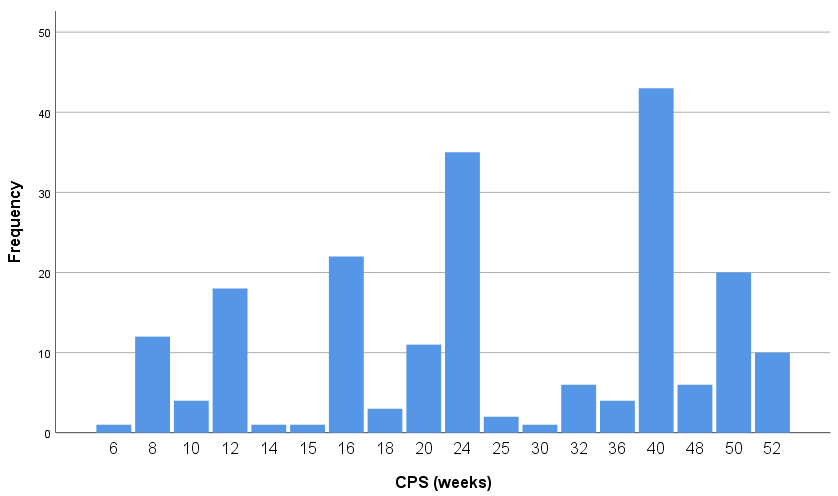

Supplement: S1 Fig — aFrequency is expressed as percentage (%). (TIF) [file pone.0267467.s001.tif]
